# Do parent–child acculturation gaps affect early adolescent Latino alcohol use? A study of the probability and extent of use

**DOI:** 10.1186/1747-597X-8-4

**Published:** 2013-01-24

**Authors:** Ronald B Cox, Martha Zapata Roblyer, Michael J Merten, Karina M Shreffler, Kami L Schwerdtfeger

**Affiliations:** 1 Human Development and Family Science, Oklahoma State University, 2403 Main Hall, Tulsa, OK 74106, USA; 2 233 Human Sciences, Department of Human Development and Family Science, Oklahoma State University, Stillwater, OK, 74078, USA

**Keywords:** Alcohol use, Effective parenting, Mother involvement, Father involvement, Curvilinear, Negative binomial, Adolescent, Latino, Acculturation

## Abstract

The literature has been mixed regarding how parent–child relationships are affected by the acculturation process and how this process relates to alcohol use among Latino youth. The mixed results may be due to, at least, two factors: First, staggered migration in which one or both parents arrive to the new country and then send for the children may lead to faster acculturation in parents than in children for some families. Second, acculturation may have different effects depending on which aspects of alcohol use are being examined. This study addresses the first factor by testing for a curvilinear trend in the acculturation-alcohol use relationship and the second by modeling past year alcohol use as a zero inflated negative binomial distribution. Additionally, this study examined the unique and mediation effects of parent–child acculturation discrepancies (gap), mother involvement in children’s schooling, father involvement in children’s schooling, and effective parenting on youth alcohol use during the last 12 months, measured as the probability of using and the extent of use. Direct paths from parent–child acculturation discrepancy to alcohol use, and mediated paths through mother involvement, father involvement, and effective parenting were also tested. Only father involvement fully mediated the path from parent–child acculturation discrepancies to the probability of alcohol use. None of the variables examined mediated the path from parent–child acculturation discrepancies to the extent of alcohol use. Effective parenting was unrelated to acculturation discrepancies; however, it maintained a significant direct effect on the probability of youth alcohol use and the extent of use after controlling for mother and father involvement. Implications for prevention strategies are discussed.

## 

During the past two and a half decades, ethnicity and culture have emerged as important moderators of risk and resilience in the etiology of Latino youth substance use. Considerable attention has been given to the relationship between acculturation to the United States and adolescent substance use [1]. However, findings regarding this association have been inconclusive. A closer look at the literature suggests the need to examine acculturation-related variables such as parent–child acculturation discrepancies in tandem with family processes implicated in the etiology of youth substance use, e.g., effective parenting and parental involvement [2,3]. In the present study, we begin to address this gap in the literature by testing a model in which the effect of parent–child differential acculturation on youth alcohol use is mediated by effective parenting and mother and father involvement. We focus on Latino youth due to the overall growth of the Hispanic population in the United States [4]; the significant portion of U.S.-born Latino youth who live with immigrant parents [5]; the reported higher rates of substance use, including alcohol, among Latino early adolescents compared to youth of the same age from other ethnicities [6]; and the reported link between acculturation and Latino substance use [7]. Alcohol use is of particular importance because alcohol is the most widely used mood altering substance among youth, and there are numerous health risks associated with underage drinking [8].

## Acculturation and Latino youth substance use

Different indicators of U.S. acculturation, such as nativity status [9], generational status [10], length of residency [11], and English acquisition [12] have been identified as strong predictors of substance use across different Hispanic groups. Nevertheless, the acculturation processes that place Latino youth at risk for substance use in the United States are still unclear. Because studies have indicated that members of immigrant families adopt the language and culture of a new country at different paces with youth generally acculturating more rapidly than adults [13], researchers seeking to explain the link between acculturation and substance use have explored parent–child acculturation discrepancies as a potential explanatory variable. Some studies conducted with Latino immigrant families have found that parent–child acculturation discrepancies place immigrant youth at risk for substance use by increasing cultural and normative conflict, reducing the family support available to children, and decreasing overall parental involvement [14-17]. Other studies, however, have found parent–child acculturation discrepancies due to children being more acculturated to the U.S. than their parents to be unrelated to family distress, parent–child conflict, or youth psychosocial adjustment [18-20].

This second group of acculturation researchers has measured acculturation in ways that allow for the child or the parent to be more acculturated than the other. This is a departure from previous studies that generally assumed that children were always more acculturated than parents, and that parents were more enculturated (oriented toward their culture of origin) than their children. Measurement that accounts for acculturation discrepancies to fluctuate in both directions for children and parents better reflect processes of staggered or serial migration common among Latinos settling in new arrival destinations [21,22]. In staggered migration, one or both of the parents immigrate first and send remittances to support their children back home. Once it is financially or legally feasible, parents bring children to live with them and reunite the family. Although at some point children may surpass their parents in their level of acculturation, staggered migration suggests that at least for a given period, some parents may be more acculturated than their children.

Studies that compute actual parent–child acculturation discrepancies and do not presume higher child acculturation produce more nuanced results and hold promise to disentangle the effects of acculturation on family processes and youth substance use. Consequently, in the present study we calculated a parent–child acculturation gap such that either the child or the parent could be highly acculturated and model a quadratic acculturation term that tests the effects of either extreme. We hypothesize that acculturation discrepancies have direct and indirect effects on youth alcohol use (i.e., probability of use and extent of use).

## Factors affecting family acculturation in new arrival states

To understand the relationship between acculturation and substance use among Latinos, studies also need to take into account the diversity of contexts in which they live. The majority of the studies investigating the link between acculturation and Latino substance use have used samples from long-standing Hispanic enclaves such as Texas, California, Arizona, and Florida. Few studies involve Latino immigrants living in new arrival states [23]. Nevertheless, the social and cultural conditions of new arrival destinations offer a particular milieu to the relationship between parenting practices and youth substance use.

There is evidence that new immigrants face significant obstacles to access the basic services (e.g., transportation, ESL classes, and stable jobs food stamps, childcare subsidies, Temporary Assistance for Needy Families) they need to sustain their families [24-27]. The limited economic and social resources available to new immigrants may impact their ability to parent effectively and be involved in their children’s schooling. New arrival states and cities are often challenged in their ability to meet the needs of Latino immigrants and their children. For example, school districts in new arrival destinations frequently lack the service infrastructure to support parental involvement among Spanish-speaking families [28-30].

Lastly, Latino immigrants settling in communities that have been linguistically, and often ethnically, homogeneous are likely to experience heightened barriers to adaptation, such as language difficulties and discrimination. From an ecological perspective [31], these contextual influences suggest that acculturation processes among immigrant families in new arrival communities differ from those in families living in established Latino enclaves [32].

## Effective parenting and youth substance use

Baumrind [33,34] reported that authoritative parenting, a style characterized by high levels of parental responsiveness (e.g., a nurturing, open communication, flexibility) and firm control (e.g., clear expectations regarding behavior), promotes higher adolescence competence and protects youth from substance use. A robust body of literature has linked parenting practices associated with authoritative parenting with older chronological onset and less frequent substance use. Some of these practices are: parental monitoring [35-37]; nurturance, warmth, and positive regard [38-41]; parental expectations and consistent discipline [42-44]; open communication [45,46]; low-conflict parent–child relationships [47,48]; and parental behavioral involvement and connectedness [35,41,49]. These parenting behaviors have been termed “effective” parenting, because they generally promote psychosocial adjustment and protect youth from substance use across a wide range of demographic characteristics. For instance, Amato and Fowler [50] found that the benefits of effective parenting extend to youth from all ethnic and socioeconomic backgrounds and family structures. Based on the body of literature supporting the generalizability of effective parenting across diverse families, we hypothesize that effective parenting has direct effects on Latino youth alcohol use, and that it mediates the relationship between parent–child acculturation discrepancies and youth alcohol use.

## Father and mother involvement and youth substance use

Parental involvement is a multidimensional construct, and comprises several domains of children’s lives. According to Grolnick and Slowiaczek [51], parental involvement is “…the dedication of resources by the parent to the child within a given domain,” a conceptualization that takes into account parents’ choices about allocation of resources to different aspects of their children’s lives, such as schooling. Hill and Tyson [52] differentiate between three types of parental involvement in schooling, 1) home-based (e.g., helping child with homework), 2) school-based (i.e., attending parent-teacher conferences), and 3) academic socialization (e.g., discussing grades with child). In the present study, we focus on parental involvement in the child’s schooling, and measure the three aspects of parental involvement identified by Hill and Tyson.

There is evidence that parental involvement in a child’s schooling indirectly influences youth substance use. For instance, research has shown that parental involvement is associated with student academic competence [53,54], and academic competence has been shown to be inversely related to substance use [55,56]. That is, youth whose parents are involved in their schooling tend to do well academically and, in turn, they are less likely to use substances. There is a paucity of studies, however, examining the direct relationship between parental involvement in schooling and youth substance use. Researchers generally have grouped various dimensions of parental involvement (e.g., behavioral, emotional) and several domains (e.g., child’s schooling, personal life) into composite scales, making it challenging to establish the unique contributions of each aspect of parental involvement to youth psychosocial outcomes [57,58]. For instance, Pilgrim, Schulenberg, O’Malley, Bachman, and Johnston [59] studied parental involvement and youth substance use using data from the Monitoring the Future study, a nationally representative sample of 8^th^, 10^th^, and 12^th^ grade students. The authors measured parental involvement using four items, two of which referred to parental help with homework. They found that parental involvement was inversely related to substance use in youth of both sexes from diverse ages and ethnic backgrounds.

It is important to further examine how parental involvement in children’s schooling influences youth alcohol use due to implications for youth development and prevention programming. Specifically, interventions that focus on parental involvement in schooling pose fewer obstacles and are likely to encounter less resistance from families than traditional approaches that openly emphasize the prevention of substance use [60]. In the present study, we define parental involvement as parental allocation of resources (e.g., time, cognitive and emotional resources) to child’s schooling in the three domains proposed by Hill and Tyson [52], i.e., home-based, school-based, and academic socialization. We hypothesize that parental involvement in child’s schooling is a mediator of the effects of acculturation discrepancies on youth alcohol use. We further discriminate between the effects of mother and father involvement on the variable of interest.

## Differences between mother and father involvement

Studies that have included families from diverse backgrounds indicate that fathers as well as mothers make unique contributions to children’s psychosocial adjustment. For example, Amato and Rivera [61] utilized the National Survey of Families and Households to examine the contribution of father involvement (reported by fathers) for children’s externalizing behaviors (reported by mothers), controlling for mother involvement. The authors found that both father and mother involvement were significantly and additively associated with lower externalizing behaviors, and that the protective effects of father involvement held across family structures (i.e., biological fathers and stepfathers) and ethnic backgrounds (i.e., Caucasian, African American, and Hispanic).

Still, there are few studies examining the distinct effects of mother and father involvement in psychosocial outcomes among Latino youth [62]. When studies have distinguished between mother and father involvement, significant differences in youth’s perceptions and their effects on the variables of interest often have been found. For instance, Paulson and Sputa [63] reported that adolescents (and parents) perceived mothers to be more involved in schooling at home (e.g., helping with schoolwork) and at school (e.g., attending school events) than fathers, although youth did not perceive differences between mothers and fathers in academic socialization (e.g., expectations about academic achievement). Kim and Rohner [64] reported that father involvement, but not mother involvement, mediated the relationship between father warmth and academic achievement in a sample of Korean families.

We found no studies that address the distinct contributions of mother and father involvement on youth substance use in the context of acculturative processes common among Latino families. Because of the potentially important and distinct contributions that mothers and fathers may have in the lives of Latino youth, in the present study we test for the unique effects that mother and father involvement in youth’s schooling may have on youthful alcohol use. Due to the lack of a literature reporting on how mother and father involvement might differentially affect child substance use, we do not present a directional hypothesis and leave this question as exploratory.

## Modeling substance use

Many studies on youth substance use do not distinguish how predictor variables might be related differently to the onset of substance use and extent of use for those who have started using. Insight into how these different elements relate to acculturation and substance use is an important step in clarifying the mixed findings in the acculturation and substance use literature. In the present study, we use an approach that models past 12-month substance use as an ordinal count variable with a zero-inflated negative binomial distribution. Modeling substance use in this way allows for a more nuanced examination of how parental and cultural variables are related to different aspects of substance use by simultaneously estimating both the probability of having used during the past year and the extent of use for those who have used.

## Methods

### Sampling procedures

Participants were seventh grade students from twelve schools in an urban school district in the Midwestern United States. All students present and willing to participate the day of data collection in May 2009 were surveyed (*N* = 1,736; 98% participation rate). Data were collected over a two-week period in May of 2009 using standardized self-report surveys in English or Spanish. Questions were read to students to avoid confounding due to literacy and to help maintain children on task. Students with learning disabilities severe enough to be exempt from annual end-of-instruction exams were excluded from the study.

Oklahoma State University’s Office of Research Compliance and the school district’s Planning, Research, and Evaluation Department granted permission to conduct the study, which included approved consent and assent forms. Additionally, permission to conduct the study was obtained from the school district and the principal of each school sampled.

### Participants

Participants for the current study were all students who self-identified as Latino(a) (*n* = 631) from the larger sample of 1,736 participants. Mean age of the Latino participants was 13.14 years, and 47% were female. The sample was primarily low-income with over 95% receiving free or reduced lunch; 57% reported living with both biological parents. Missing values for the study variables were low ranging from .1% to 4%. Missing values were handled using Full Information Maximum Likelihood estimation in Mplus v6.0 [65].

### Measures

#### Past 12-month alcohol use

Alcohol use was measured by a single item, “During the last year, how often did you drink alcohol?” Responses were made on an eight-point scale ranging from 0 (*never*) to 7 (*once or more per day*).

#### Mother and father involvement

**Figure 1:**
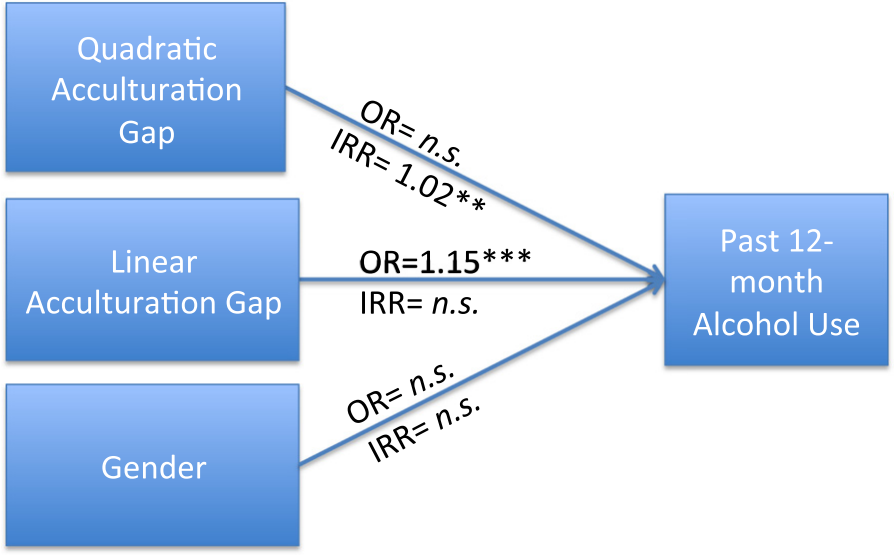
**Direct effect of Acculturation Gap on Past 12 month Alcohol Use Controlling for Gender.** (*p<.05. **p<.01. ***p<.001, n.s. = not significant). OR = Odds Ratio, and IRR = Incidence Rate Ratio. Significance tests use the Wald test, which is the ratio of the estimated regression coefficient, divided by its standard error, evaluated with a *t* or *Z* test [76].

Mother and father involvement were each measured by a latent construct with five items that capture aspects of parental involvement in the child’s schooling (e.g., *My mother or mother figure: 1) makes sure I do my homework, 2) discusses report cards with me; 3) attends parent-teacher conferences*). Items for the latent construct were placed on a four-point scale ranging from strongly disagree to strongly agree so that higher scores indicate more involvement. In the current study, measures of internal consistency for mother and father involvement were strong (α *=* .82, and .89) respectively.

#### Effective parenting

Effective parenting was measured by a latent construct with four items adapted from measures of parental care and support used in prior survey research with adolescents [66]. The items were designed to capture adolescent’s perception of effective parenting as represented by level of parent–child relationship conflict, parental positive regard for child, parental behavioral monitoring, and communication. Sample items are: *My parents/guardians know how I think or feel about things important to m*e, and *We often have arguments that end in fights*. Items for the latent construct were placed on a four-point scale ranging from strongly disagree to strongly agree so that higher scores indicate more effective parenting. Internal consistency for the four items was adequate (α = .65).

#### Parent–child acculturation discrepancy (Gap)

This variable was created using youth perceptions of self and parents’ language proficiency (e.g., *How well does your Mother/Father speak English?*) as rated on a 5-point Likert scale*.* We chose language proficiency as our measure of acculturation because English language proficiency has been consistently associated with substance use among Latino groups in the U.S. [67], and is considered a robust measure of acculturation accounting for up to 65% of the variance in acculturation status [68,69]. The discrepancy score or “gap score” was created by: 1) averaging the mother and father English proficiency to create a parent scale; 2) standardizing the youth scale; 3) standardizing the parent scale on the same scale by using the youth mean and standard deviation; and 4) subtracting the parent score from the youth score. In this way higher (positive) scores reflect the child being more linguistically acculturated than the parent, a zero score signifies equal levels of acculturation, and lower (negative) scores indicate the parents being more linguistically acculturated than the child.

### Analytic plan

A structural equation model tests the association of parent–child acculturation discrepancy on alcohol use during the past year. The outcome variable is ordinal, which we treat as a count variable. When numerous participants do not indicate any previous 12-month use, this variable is called zero-inflated [70]. In our data, a preponderance of participants (62.4%) indicated no use of alcohol during the past 12 months. Since a zero-inflated negative binomial model (ZINB) can be considered nested within a standard negative binomial model, the zero inflation assumption was tested using a Chi-squared difference test. Results indicate a significant difference between the two models for alcohol use, so we proceed with the ZINB model. The ZINB model allows us to simultaneously estimate two regressions. First, a logistic regression predicts the probability of being in the true nonuse category (i.e., a latent class of individuals who would never use that drug that year). The second regression uses a negative binomial distribution to predict the frequency or extent of use among the latent class of those who use alcohol, including users estimated to use it zero times according to the negative binomial distribution. It should be noted that both the “probability of use” and the “extent of use” resulted from the same original substance use variable. Path coefficients to predict the binary portion of the outcome variable are translated into odds ratios (*OR*) or the percentage increase in the odds of use of alcohol given a one-unit increase in the covariate. Likewise, path coefficients to the count portion of the outcome variable are understood as incidence rate ratios (*IRR*) or the percentage increase in the odds of increasing the expected count by one for alcohol use given a one-unit increase in the covariate. The null hypothesis used to understand *OR* and *IRR* values is 1.00, with values under 1 indicating a negative association and values over 1 indicating a positive association.

Confidence intervals for the indirect effects were constructed using PRODCLIN [71]. Indirect effects of acculturation (i.e., linear and quadratic terms) on the likelihood of use and on the extent of use of alcohol were tested for significance using MacKinnon’s asymmetric distribution of products test [71]. This procedure was chosen because it can test more than one mediating sequence at a time, and the direct relationship does not have to be statistically significant for mediation to exist. The asymmetric distribution of products test constructs a confidence interval around the product of the two unstandardized path coefficients that make up mediated relationship (i.e., an outcome regressed on an exogenous variable X through a mediator). Because our analyses included dichotomous and count dependent variables that were reported as OR and IRR, exponentiated confidence intervals that did not include the value of 1.00 indicated significant mediation [71]. Significant indirect relationships were regarded as full mediation if the direct effects of the acculturation terms were also no longer significant and partially mediated if the direct effects remained significant after controlling for the mediating variables [72].

Because the sample is nested among 12 schools, we adjust for the non-independence due to clustering in the data using the Type=Complex command in Mplus. The Type=Complex command in Mplus adjusts the standard errors using full information maximum likelihood estimation but does not allow for modeling of level-two variables. Mplus v.6 [65] is used to estimate the ZINB model with full information maximum likelihood with robust standard errors.

### Model building

Following the two-step modeling approach recommended by Gerbing and Anderson [73], we tested a measurement model of the hypothesized latent variables before evaluating our structural path models of interest. Confirmatory factor analysis was used to test the factor structure of the latent constructs. With large samples, adequate fit between the sample and fitted covariance matrices is indicated by a normed Chi square (χ^2^ model/*df*) ≤ 5 [74], CFI > .95, TLI > .95, and/or a RMSEA ≤ .06 [75]. The measurement model including effective parenting, mother involvement, and father involvement fit the data adequately, (χ^2^ [78] = 172.10, *p* < .001, CFI = .98, TLI = .97, RMSEA = .04). Factors loadings for the latent constructs are shown in Table 1.

**Table 1:** Confirmatory factor analysis factor loadings

	**Loading**	**t-Value**
*Mother Involvement*		
Make sure homework done	0.71	29.61
Praise for study and grades	0.71	29.70
Aware of attendance	0.64	24.01
Discuss report card	0.80	41.91
Attend conferences	0.55	17.66
*Father Involvement*		
Make sure homework done	0.82	50.38
Praise for study and grades	0.78	42.76
Aware of attendance	0.78	41.49
Discuss report card	0.84	54.44
Attend conferences	0.58	20.49
*Effective Parenting*		
Aware of what is important to child	0.68	24.57
Know child’s whereabouts	0.64	22.06
Arguments often end in fights	−0.39	−10.20
Child feels important	0.65	22.29
Warn about drugs and alcohol	0.66	23.38

**Figure 2:**
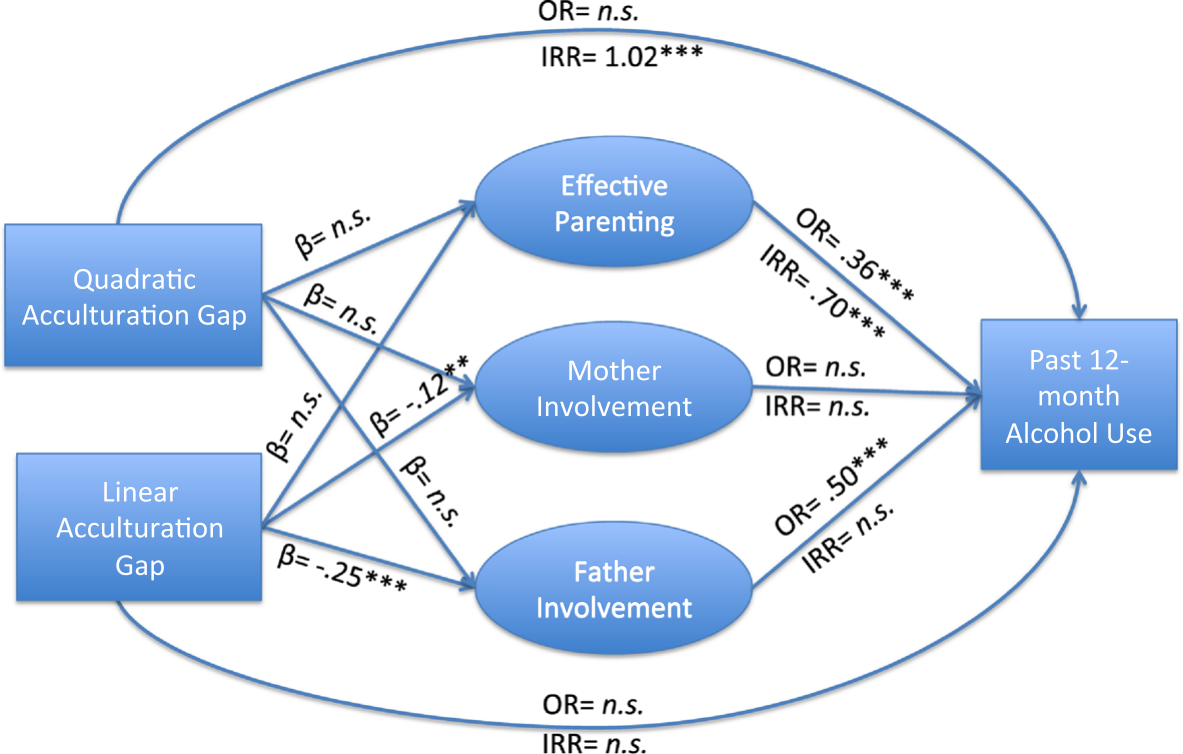
Effect of Acculturation Gap on Past 12 month Alcohol Use via Effective Parenting, Mother Involvement and Father Involvement(*p<.05. **p<.01. ***p<.001, *n.s. *= not significant).*ß* = Standardized Regression Coefficient, *OR* = Odds Ratio, and *IRR* = Incidence Rate Ratio. Correlation coefficients for the mediators (not shown in the model) are: Mother Involvement with Father Involvement (r =.62, p<.001); Effective Parenting with Father Involvement (r =.47, p<.001); Effective Parenting with Mother Involvement (r =.72, p<.001). Significance tests use the Wald test, which is the ratio of the estimated regression coefficient, divided by its standard error, evaluated with a *t* or *Z* test [76].

Next, a structural path model was used to simultaneously test direct and indirect associations among the study constructs. Our hypothesized models are shown in Figures 1 &2. The structural mediational model was implemented following a two-step approach. First, we modeled direct paths of acculturation discrepancy and gender on the probability of having used and the extent of use among users for past 12-month alcohol use without mediators; and second, we added the mediated paths to the models (see Table 2 for path coefficients with confidence intervals and corresponding Wald tests).

**Table 2:** Coefficients, test statistics, degrees of freedom and confidence intervals for path models within the structural equation model

	**Direct Effects Model**					**Mediation Model**				
**Outcome Predictors**	**Coefficient**	*t*-value*	***df***	**95% CI**		**Coefficient**	*t*-value*	***df***	**95% CI**	
*Probability of alcohol use on*	OR			Lower	Upper	OR			Lower	Upper
Quadratic acculturation gap	1.01	0.66	627	0.98	1.05	0.998	−0.05	625	0.93	1.07
Linear acculturation gap	1.15	3.39	627	1.06	1.25	1.10	1.93	625	1.00	1.22
Gender	1.14	0.74	627	0.80	1.64					
Effective parenting						0.36	−3.12	625	0.19	0.68
Mother involvement						0.99	−0.02	625	0.41	2.42
Father involvement						0.50	−3.33	625	0.33	0.75
*Extent of alcohol use on*	IRR					IRR				
Quadratic acculturation gap	1.02	2.71	627	1.003	1.02	1.02	2.93	625	1.01	1.03
Linear acculturation gap	1.02	0.43	627	0.94	1.10	1.04	1.08	625	0.97	1.12
Gender	1.07	0.74	627	0.90	1.27					
Effective parenting						0.70	−3.35	625	0.57	0.86
Mother involvement						0.98	0.12	625	0.71	1.36
Father involvement						0.85	1.93	625	0.73	1.00
*Effective parenting on*						β				
Quadratic acculturation gap						−0.04	−0.55	628	−0.18	0.10
Linear acculturation gap						0.04	0.65	628	−0.08	0.15
*Mother involvement on*										
Quadratic acculturation gap						−0.04	−1.09	628	−0.12	0.04
Linear acculturation gap						−0.12	−2.97	628	−0.20	−0.04
*Father involvement on*										
Quadratic acculturation gap						−0.10	−1.83	628	−0.20	0.01
Linear acculturation gap						−0.25	−7.96	628	−0.32	−0.19

*Note.* OR = Odds Ratio, IRR = Incidence Rate Ratio, CI = Confidence Interval. *We use the Wald test, which is the ratio of the estimated regression coefficient, divided by its standard error, evaluated with a *t* or *Z* test [76]. Reported degrees of correspond to the Wald test and not to the chi-square test of fit for the structural equation model.

## Results

### Direct effects (Figure 1)

#### Parent–child acculturation discrepancy

For interpretability, acculturation gap was mean centered (*M* = 1.66) for all analyses and then squared to test the quadratic effect. Descriptive statistics show that approximately 7% of parents (average of mother and father) in our sample spoke English better than their child, 62% spoke English worse than their child, and 31% were identical in their scores. Of non-identical scores, about 2.7% favored the parents by more than one standard deviation and about 26% favored the child by more than one standard deviation. Thus, there were acculturation gaps in both directions, although children usually spoke English better than the average of their parents.

We first estimated a direct effects model that included parent–child acculturation discrepancy (acculturation gap) and the quadratic term of the same (acculturation gap^2^) controlling for youth gender. Holding gender constant, a linear increase in acculturation gap was significantly associated with the probability of using but not the extent of use for alcohol. Acculturation gap^2^ (the quadratic term) was not associated with the probability of having used alcohol during the past 12 months, but was significantly associated with increased extent of use as indicated by the incident rate ratio (*IRR*).

#### Youth gender

With acculturation gap and acculturation gap^2^ in the model, youth gender was not a significant predictor of either the probability of being in the user group or the extent of alcohol use during the previous 12 months. Therefore, it was dropped from the mediation model for the sake of parsimony.

### Mediation model (Figure 2)

Next, we added the three hypothesized mediators to the model (i.e., mother involvement, father involvement, and effective parenting). We hypothesized that effective parenting and mother and father involvement in youth’s schooling would mediate the path from acculturation discrepancy to the probability of having used alcohol and the extent of use for users during the past 12 months. In general, we found support for the mediation effects of father involvement, but not for effective parenting or mother involvement.

#### Effective parenting

Effective parenting was not significantly predicted by either acculturation gap term. It was, however, significantly and negatively associated with the probability of using, and with a decrease in the extent of alcohol use during the past 12 months. That is, a one unit increase in effective parenting decreases the probability of using alcohol within the last year by 64% and the extent of use by one point by 30% among users. Effective parenting did not mediate the path from acculturation gap or acculturation gap^2^ to either the probability of using or to the extent of alcohol use according to PRODCLIN [71].

#### Father involvement

Father involvement was significantly predicted by the linear acculturation gap, but not by the quadratic acculturation gap. That is, for a standard deviation increase in the child being more acculturated than the parents there was a .25 standard deviation decrease in father involvement in youth’s schooling. Father involvement significantly predicted the probability of using, but did not predict the extent of use among users. That is, each unit increase in father involvement in the child’s schooling decreases the probability of using alcohol during the past year by 50%. Father involvement significantly mediated the path from acculturation gap to the probability of using alcohol (OR = 1.09, 95% CI = 1.03, 1.15) but did not significantly mediate the path to extent of use for those who reported using during the previous 12 months. Father involvement did not mediate the path from acculturation gap^2^ to either the probability or extent of past 12-month alcohol use.

#### Mother involvement

Mother involvement was significantly predicted by the linear acculturation gap, but not the quadratic acculturation gap. That is, a one standard deviation increase in the child being more acculturated than the parents results in a .12 standard deviation decrease in mother involvement in the child’s schooling. Mother involvement was not associated with either the probability of using or the extent of alcohol use, nor did it mediate the path from acculturation gap or acculturation gap^2^ to either the probability of using or to the extent of alcohol.

#### Parent–child acculturation discrepancy and alcohol use

After adding the mediators to the model, the linear gap term no longer directly increased the probability of use significantly, and the quadratic gap term remained a significant predictor of the extent of alcohol use. Thus, the linear effect of acculturation gap on the probability of alcohol use was fully mediated by father involvement and the quadratic effect of acculturation gap^2^ on the extent of use was not mediated by either effective parenting or father or mother involvement.

## Discussion

This study examines the unique effects of parent–child acculturation discrepancies (gap), mother involvement in children’s schooling, father involvement in children’s schooling, and effective parenting on youth alcohol use during the last 12 months, measured as the probability of using and the extent of use. Direct paths from parent–child acculturation discrepancy and youth gender to alcohol use, and mediated paths through mother involvement, father involvement, and effective parenting were tested.

### Direct effects

**Figure 3:**
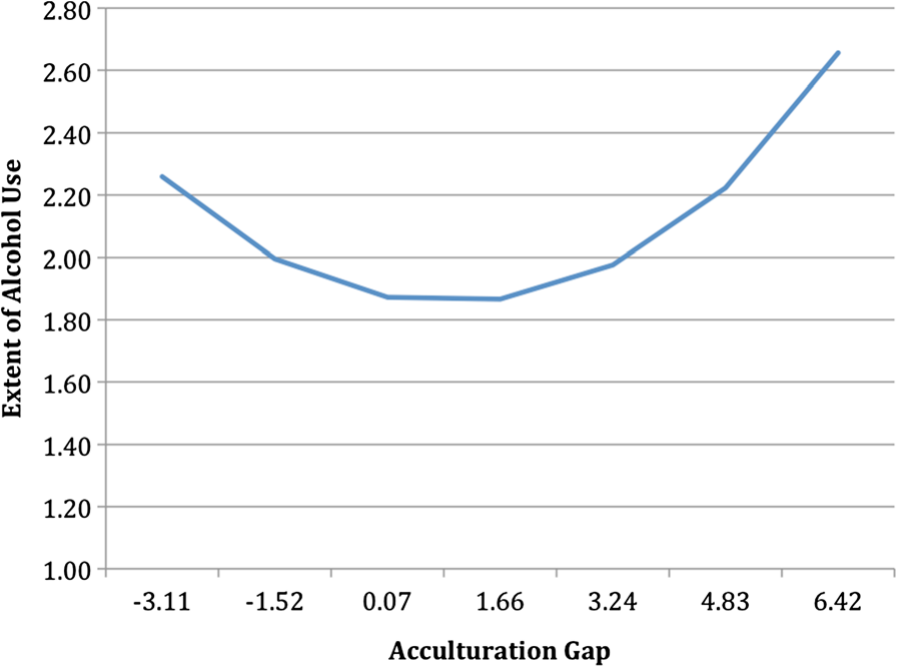
Curvilinear Effect of Acculturation Gap on the Extent of Alcohol Use.

The literature has been somewhat mixed regarding how acculturation is related to alcohol use among Latino youth. The mixed results may be due to, at least, two factors considered in the present study: First, acculturation may be related differently to the probability of using alcohol than to the extent of use among users. Second, the acculturation process can occur faster in either parents or children, which this study investigated by testing for curvilinear effects. In the present study the linear effect of the acculturation gap is significantly related to the probability of using (albeit modestly), but not the extent of use. The quadratic effect of the acculturation gap is significantly related to the extent of use, but not with the probability of having used. The mean value of the acculturation gap is 1.66, indicating that children in our sample are about one standard deviation more linguistically acculturated than parents on average (see Figure 3).

These findings suggest that acculturation gap (youth more linguistically acculturated than parents) modestly increases the odds that youth have used alcohol at least once in the 7^th^ grade. Among pre-adolescents who have used alcohol at least once, however, there is a curvilinear association of the acculturation gap on their frequency of drinking (See Figure 3, showing the curvilinear effect). This curvilinear effect suggests that it is predominately youth who know English much better than their parents who are at risk of drinking substantially more than youth who are more similar to their parents in linguistic acculturation. Our data suggest that 7^th^-grade Latinos who drink alcohol do so less frequently when they are somewhat similar to their parents in English proficiency. The significant curvilinear effect suggests that acculturation gaps in either direction put youth at risk for excessive drinking, but we do not have a sufficient sample of youth who know English far less than their parents to reliably make this determination (only 7% of parents in our sample spoke more English than their children). Rather, it seems that youth who are somewhat similar to their parents in linguistic acculturation are at little risk of excessive drinking, but those who know English much better than their parents are more likely to drink excessively if they are in the user group. Further, the linear effect of the gap also indicates that they are at the most risk of being in that user group.

This risk of being in the user group is mediated, however, by father involvement; the linear effect of the acculturation gap reduces the school involvement of both mothers and fathers, but father involvement with schools reduces the chance that youth will be in the user group, unlike mothers’ involvement. Once the youth are using alcohol, however, father involvement does not reduce the extent of excessive drinking.

In contrast to other studies [77], our findings do not show that effective parenting mediates the path from acculturation gap to alcohol use. However, effective parenting does act independently to greatly reduce the youth’s likelihood of using alcohol and, if they drink, it substantially reduces the extent of drinking.

To our knowledge this is the first study to empirically test for a curvilinear relationship between alcohol use and acculturation and may help clarify some of the mixed findings in the literature. Previous studies have found small or no effects for acculturation [1], which would be expected if a curvilinear effect were present. Because a linear test fits the best fitting straight line to the data it contrasts parent-greater acculturation vs. child-greater acculturation, and misses the intermediate scores of acculturation (parents and children equally acculturated) that were associated with lower rates of substance use in our data. Once the possibility of differential acculturation in both directions is acknowledged, a possible hypothesis is that differential acculturation in either direction will put the child at greater risk for substance use. As shown in Figure 3, our results support that hypothesis to some degree, although we cannot be confident in how widespread that effect might be until differential acculturation in both directions is investigated in other samples. In addition, our curvilinear test is consistent with the conclusion that it is extreme differential acculturation that is the major risk factor, and that small degrees of differential acculturation do not put children at risk of substance use. For both reasons testing for a curvilinear effect should be a standard procedure in this body of research. Depending upon the accompanying linear effect, a curvilinear effect may indeed discover that both extremes of acculturation discrepancy are more positively associated with substance use than are intermediate values. Without the quadratic term there is no way to discern whether the effect of parents being more acculturated than their children puts children more at risk in addition to the most common type of differential acculturation.

### Indirect effects

#### Effective parenting

Consistent with past research, we found that increases in effective parenting are associated with decreases in alcohol use. We also found no evidence of a relationship between acculturation gaps and effective parenting. These findings may be related to our sample being from a geographical location where Latino immigration is relatively recent.

Other researchers have noted that immigrant families often value their children’s cultural adaptation, particularly in the form of language acquisition [78]. Learning the host country’s language positions children as esteemed cultural brokers [79] that are crucial to the success of the whole family [80] and may lessen the negative impact of acculturation discrepancies on intergenerational parent–child relationships. Some studies indicate that children of immigrants often assist their parents with job and financial responsibilities, either working alongside them or earning money the family comes to rely upon for economic subsistence [81]. In this context, more acculturated children are highly esteemed as contributing members of the family, which may increase their sense of belonging and attachment to the family and decrease the impact of acculturation gaps.

Given the mixed findings in the literature regarding the role of effective parenting in the relationship between parent–child acculturation discrepancies and negative youth outcomes like alcohol use, it may be that context (e.g., a geographical region in which Latino immigration is relatively new as compared to more traditional destination) plays an important role. That is, the way in which effective parenting relates to acculturation discrepancies and alcohol use depends on the interaction between contextual influences and family characteristics that allow some families to use acculturation gaps to their benefit while other families are disrupted by the same. At this point, these arguments are speculative and should be tested empirically to help clarify discrepant findings in the literature on how intergenerational acculturation discrepancies affect Latino youth outcomes.

#### Father involvement

Father involvement is associated with the linear acculturation gap such that a one standard deviation increase of child being more acculturated than parents is associated with a .25 standard deviation decrease in father involvement in child schooling. Increases in father involvement are also associated with decreases in the probability of having used but not in the extent of use among users. Father involvement also mediates the relationship between the linear acculturation gap and the likelihood of use.

These findings support the importance of father involvement as a protective factor for the onset of substance use and suggest that promoting father involvement in schooling may be an effective strategy to prevent or delay substance use among Latino youth without focusing specifically on substance use behaviors. Previous studies have indicated that familial protective factors against substance use exert their strongest effect on exposure to the substance [82,83]. That is, father involvement influences youth alcohol use through reducing opportunities to use.

#### Mother involvement

After controlling for father involvement and effective parenting, mother involvement was not associated with the probability or extent of alcohol use and did not mediate any of the relationships. The most parsimonious explanation for these findings may be that most youth in the study perceived their mothers as highly involved. Several studies have indicated that adolescents see their mothers as more involved than their fathers [63,84]. This generalized perception would lead to attenuated statistical variation in youth’s reports of mother involvement compared to father involvement [85].

### Limitations

This study has several limitations. First, our study only includes students in seventh-grade classrooms in an urban school district in the Midwest, and thus findings should not be generalized to the larger Latino population. Similarly, seventh graders are in early adolescence and may not have had as many opportunities to become involved with extensive use of alcohol. It is likely that our findings will vary somewhat for youth in middle or late adolescence. Second, although language is an established and often used proxy for acculturation, a more comprehensive multidimensional measure of acculturation might have yielded different results. Third, because a majority of our sample are first and second generation Latino immigrants with low economic resources (approximately 95% are on free and reduced lunch) we did not include parental education or SES in the models. It is plausible that parents with higher levels of education and more economic resources may be more inclined to be involved in their child’s schooling. Fourth, the current study does not control for intercept values. That is, the study assumes that a calculated difference score of X between parent and child acculturation holds the same meaning along the continuum of scores. We know of no other studies on acculturation that have controlled for intercept values and therefore cannot estimate how this affects the current findings. Further research is needed to assess how the same difference score may vary in meaning along the continuum of scores. Finally, the same limitations inherent to all cross-sectional studies in regards to inferring causal relationships should be noted.

### Contributions and future directions

Notwithstanding the above-mentioned limitations, this study contributes to the literature in several ways. First, measures of father involvement, mother involvement, and effective parenting allow for the comparison of the unique effects of each variable as it relates to both acculturation discrepancies and alcohol use. Fathers’ contributions to youth outcomes are particularly important because the literature has focused mostly on mothers’ parenting [86]. Moreover, father involvement among Latino families has been largely unexplored or has been approached from stereotypical views that represent Latino fathers as uninvolved in their children’s lives or as mere economic providers [87]. Our study illustrates the importance of including measures of father involvement in studies of acculturation.

Second, finding that father involvement in children’s schooling is negatively associated with the likelihood of youth alcohol use indicates that this parenting practice affects more than one domain of youth behavior. Promoting parental involvement in school could be an approach that reduces the need for multiple interventions to prevent a range of negative youth outcomes, and one that is likely to encounter less resistance from families than traditional youth-oriented programs intended to prevent youth substance use.

Third, this study adds to a growing literature emphasizing a more nuanced approach to of the study of intergenerational acculturation discrepancies and negative youth outcomes. Future studies involving Latinos in recent arrival states need to consider acculturation discrepancies due to parents being more acculturated than children, which may have very different implications for youth psychosocial outcomes than discrepancies due to greater child acculturation.

Fourth, a zero-inflated negative binomial modeling approach for high-risk behaviors in youth has the advantage of utilizing all of the data available, and provides unique insight into the effects of risk and protective factors on distinct aspects of youth behavior. This study highlights the need to measure distinct aspects of alcohol use simultaneously since experimentation and frequent use are likely to be influenced differently by contextual variables. This approach holds promise to provide more detailed information for the development of prevention strategies.

Finally, studies on adolescents indicate that alcohol use increases radically during early adolescence, from the ages of 12 through 15 years [88], and the younger the age of initiation, the more likely teens are to engage in other substance use and to develop an alcohol use disorder as adults [89]. Our study indicates that among Latino youth, effective parenting exercises a strong protective influence on whether a pre-adolescent initiates use, and adds to the burgeoning body of literature pointing to the importance of effective parenting in preventing or delaying alcohol and other substance use. On the other hand, ineffective parenting has been called one of the major public health issues of our time [90]. Although positive parenting behaviors are not a panacea for all issues youth face, components of parental involvement are increasingly being included in school-based programs to prevent alcohol and drug use with success [91,92]. For these programs to become widespread, change is needed across systems to promote family-friendly legislation and implement effective parenting education [93]. Such investments can create the conditions in which families may excel in rearing the next generation and be proactive in deterring adolescent substance use and abuse.

## Competing interests

All authors declare that there are no competing interests. None of the authors has received any financial support of any kind from an organization or institution that might gain financially from the publication of this article. There are no stocks, patents, or any other links with any other institution that might influence the findings or benefit from the publication of this article in any way. The authors are affiliated with the Oklahoma Center for Health Sciences who maintains a subscription that covers the processing fee of the article.

## Authors’ contributions

RBC was the PI on the project and worked in all aspects of the study design, data collection, analyses, literature review, and writing of the manuscript. MZR is a graduate student who contributed to the literature review and writing of the manuscript. MJM is a Co-PI on the project and worked in all aspects of the study design, data collection, and provided help in writing of the manuscript. KMS is a Co-PI on the project and worked in all aspects of the study design, data collection, and provided help in writing of the manuscript. KLS is a Co-PI on the project and worked in all aspects of the study design, data collection, and provided help in writing of the manuscript. All authors read and approved the final manuscript.
